# 

**Published:** 1886-06

**Authors:** 


### CONTENTS:

ARTICLE I—The Mucous Membrane of the Mouth. By Edwin A. Cormack, L. R. C. P. & S., L. D. S.... 49
ART. II—Probable or Possible Poisoning from Dentists' Amalgams. By a Physician 65
ART. III—Reproduction of Bone. By Joseph Kurtz, M. D. .... 73
ART. IV—Recent Progress in Dentistry. By William Herbert Rollins.... 79

### EDITORIAL, ETC.

On the Rotation Method—the Construction of the First Gold Layers.... 88
The American Dental Association .... 89
The Georgia State Dental Board.... 90

### MONTHLY SUMMARY.

Something New in Making Rubber Plates.... 91
Caries of Left Side Inferior Maxilla.... 92
Arsenic in Killing Pulps of Teeth.... 93
Tuberculosis of the Buccal Mucous Membrane.... 94
Intimacy Breeds Contempt.... 95
A Remarkable Case.... 95
The Treatment of Sick Headache.... 96

